# Technical difficulties during electronic cardiac device implantation in patients with persistent left superior vena cava

**DOI:** 10.1016/j.heliyon.2022.e09801

**Published:** 2022-06-27

**Authors:** Nika Kuridze, Kakhaber Etsadashvili, Eteri Minadze, Beka Rukhadze, Nana Bakashvili, Mikheil Tsverava

**Affiliations:** a Faculty of Clinical and Translational Medicine, Ivane Javakhishvili Tbilisi State University, Georgia; b Rhythmology Department, G. Chapidze Emergency Cardiology Center, Tbilisi, Georgia; c Outpatient Department, G. Chapidze Emergency Cardiology Center, Tbilisi, Georgia

**Keywords:** Persistent left superior vena cava, Cardiac implantable electronic device, Cardiac pacemaker

## Abstract

We report a case of persistent left superior vena cava (PLSVC), discovered by chance during the cardiac electronic device placement procedure. PLSVCs are congenital anomalies of the thoracic vasculature, during which remnants of the left superior vena cava drain into the right atrium through the coronary sinus. PLSVCs can vary in their location and overall anatomy. In patients with PLSVC, implantation of a cardiac electronic device is associated with an increased risk of technical difficulties the entire procedure.

## 1 Introduction

A typical route used for cardiac electronic device placement is an approach through the left subclavian vein. Here we seek to describe the difficulties associated with this approach in a patient with a PLSVC.

PLSVCs are congenital thoracic venous anomalies present in approximately 0.3–0.5% of overall population [1]. When the anomaly exist s veins drain into the right atrium through a connection with the coronary sinus. This is usually asymptomatic and are often incidental found during various diagnostic or therapeutic procedures. Vena subclavia approach during cardiac electronic device implantation is a good example of the latter. One of the first reports of such an incidental finding is a case documented back in 1971, describing the implantation of a single chamber pacemaker in a patient with PLSVC [2].

## 2 Сase presentation

**Figure 1:**
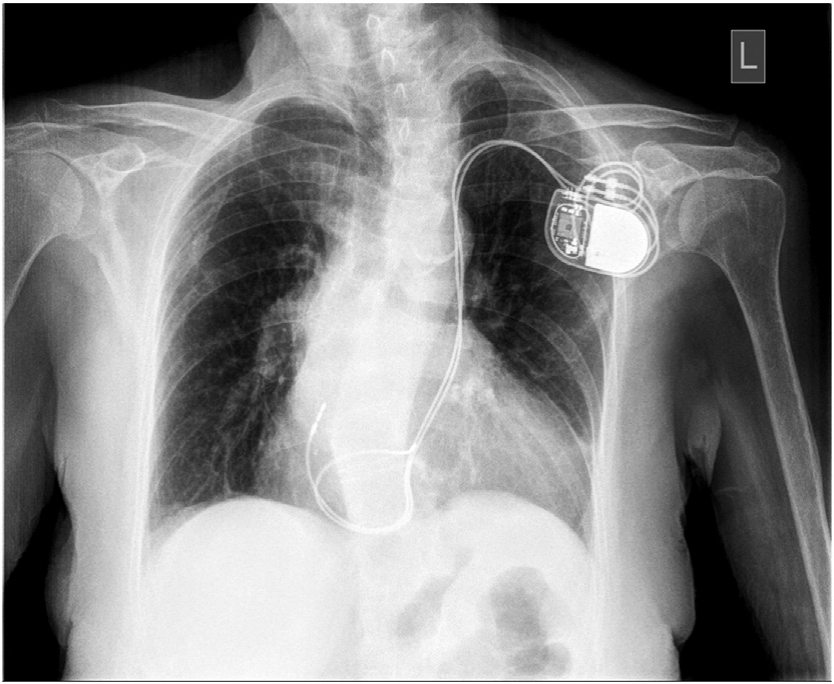
X-ray image of implanted cardiac pacemaker in patient with persistent left superior vena cava (anteroposterior view).

**Figure 2:**
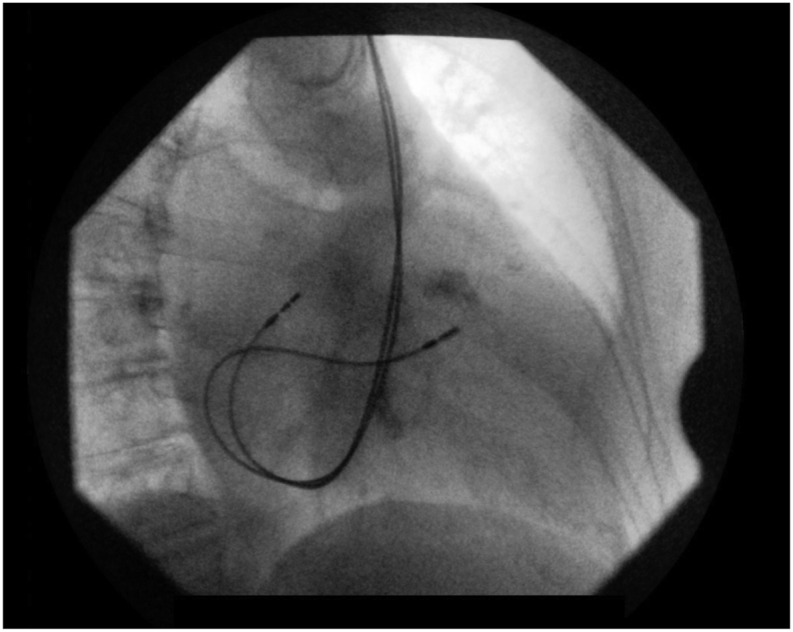
X-ray image of implanted cardiac pacemaker in patient with persistent left superior vena cava (lateral view).

Informed consent of the patient was obtained for the publication of this article. This is a case of a 65-year-old female who presented to the emergency department with complaints of general weakness, dizziness and presyncope. An urgent 12-lead ECG showed a complete AV block. After the exclusion of possible reversible causes of AV block, dual-chamber pacemaker placement was chosen as the main modality of treatment. During the procedure, approaching through the left subclavian vein, the guidewire took an unusual left- and downward course, at the level of the coronary sinus, leading to the incidental finding of PLSVC. Taking advantage of this finding, the right atrial and ventricular lead placement was attempted through an approach going through the remnant left superior vena cava and coronary sinus. The pacemaker implantation was an overall success with lead placement, using manually shaped stylets in the high right atrium and mid-septum region of the right ventricle (Figures 1 and 2).

The procedure itself, however, was complicated due to ventricular lead dislodgement during the placement, requiring repeat fixation twice. We received satisfactory results of pacemaker electrical parameters, which was sustained after 1 year later patient follow-up (Table 1).

**Table 1:** Electrical parameters, during cardiac pacemaker implantation procedure and after 1-year follow-up.

		A	V
Lead impedance [Ω]	during implantation	780	900
	after 1 year follow-up	595	820
Sensing - mean amplitude [mV]	during implantation	7,4	11,5
	after 1 year follow-up	8,0	12,0
Pacing threshold [V]	during implantation	0,9	0,8
	after 1 year follow-up	0,5	0,5

## 3 Discussion

A PLSVC is a congenital anomaly and represents the persistence of the left anterior cardinal vein, which continues into the left sinus horn (left duct of Cuvier) during the early developmental period. PLSVCs are further subclassified into different types. Most commonly, both the right and left superior vena cava are retained, with the presence of a bridging innominate vein in up to 30% of cases. According to some reports, this innominate vein is absent in up to 65% of all cases [3]. Rarest of all is the presence of PLSVC in the absence of the normal right SVC. Another uncommon finding is coronary sinus ostial atresia with the cardiac veins draining into the subclavian vein. In 80–90% of individuals, PLSVC drains into the right atrium with little to no changes in overall cardiac hemodynamics. PLSVC may also rarely drain into the left atrium, causing a right to left shunt, into the hepatic vein, or the inferior vena cava [4].

Diagnosis of a PLSVC may be obtained with a plain chest radiograph or transthoracic echocardiogram (TTE), though the definitive method is multislice spiral computer tomography (CT) or magnetic resonance venography (MRV). The PLSVC typically does not produce any symptoms [5], is found accidently during cardiac catheterization or surgery. A finding suggestive of PLSVC on TTE includes dilation of coronary sinus ostium, though this is not necessarily specific to PLSVC. Up to 40% of patients with this anomaly usually have other cardiac malformations [6], including bicuspid aortic valve, aortic coarctation, atrial septal defect, atresia of coronary sinus ostium, and cor triatriatum. PLSVC has been associated with anatomical abnormalities affecting the sinoatrial node and the overall conductive system of the heart. Fetal dispersion of the SA and AV nodes within the central fibrous body is a clear example of such an abnormality, creating a rich substrate for the initiation of arrhythmias [7].

The risks associated with electronic cardiac device placement in patients with PLSVC does not only entail technical difficulties with lead fixation. Moreover, the risks of lead misplacement, vessel injury, triggering cardiac ischemia or arrhythmia, cardiogenic shock, or cardiac arrest are all increased risk [8]. In some cases, cardiac lead placement might be impossible or may require the use of different shapes of stylets. While the placement of single- or dual-chamber devices in patients with PLSVC is difficult enough, implantation of CRT-P/D devices is even further complicated by the challenge of positioning the third lead into the left posterolateral vein of the heart through the PLSVC. Before the implantation of electronic cardiac device, a left venogram can be employed to establish the diagnosis of PLSVC. In patients with PLSVC, implantation through the right SVC is preferable from the viewpoint of procedural complexity, unless the patients developed conditions favoring a left-sided approach in the presence of right SVC, such as venous occlusion or infection of previously implanted devices. A routine left venogram might define the procedural approach and avoid possible difficulties during implantation.

Parallel to the rising number of cardiac electronic device implantations, rising transvenous lead extraction procedures. Noteworthy are the challenges we face during lead extraction in patients with PLSVC. During this type of procedure, there are technical challenges, but in experienced centers lead extraction can be performed with favorable outcomes.

## 4 Conclusion

High suspicion for the presence of a PLSVC should be present in cases where TTE clearly shows dilation of the ostium of the coronary sinus, which should lead to diagnostic testing with either CT or MRV. Patients with known PLSVC should be made aware of the increased difficulties and potential risks of cardiac electronic device implantation, though these do not necessarily constitute an absolute contraindication to the procedure itself. In the presence of the indication, implantation of electronic cardiac devices in patients with PLSVC are beneficial for general health and improved quality of life.

## Declarations

### Author contribution statement

All authors listed have significantly contributed to the investigation, development and writing of this article.

### Funding statement

This research did not receive any specific grant from funding agencies in the public, commercial, or not-for-profit sectors.

### Data availability statement

Data will be made available on request.

### Declaration of interests statement

The authors declare no conflict of interest.

### Additional information

No additional information is available for this paper.
